# Highly Pathogenic Porcine Reproductive and Respiratory Syndrome Virus Infection Induced Apoptosis and Autophagy in Thymi of Infected Piglets

**DOI:** 10.1371/journal.pone.0128292

**Published:** 2015-06-05

**Authors:** Gang Wang, Ying Yu, Yabin Tu, Jie Tong, Yonggang Liu, Chong Zhang, Yafei Chang, Shujie Wang, Chenggang Jiang, En-Min Zhou, Xuehui Cai

**Affiliations:** 1 State Key Laboratory of Veterinary Biotechnology, Harbin Veterinary Research Institute, Chinese Academy of Agricultural Sciences, Harbin, 150001, PR China; 2 Department of Preventive Veterinary Medicine, College of Veterinary Medicine, Northwest A&F University, Yangling, Shaanxi, 712100, PR China; 3 Jilin Agricultural University, Changchun 130118, PR China; University of Hong Kong, CHINA

## Abstract

Previously, we demonstrated that the highly pathogenic porcine reproductive and respiratory syndrome virus (HP-PRRSV) HuN4 strain causes obvious thymic atrophy and thymocytes apoptosis in infected piglets after birth, which is more severe than that induced by classical PRRSV. In this study, we investigated apoptosis and autophagy in the thymus of piglets infected with the HP-PRRSV HuN4 strain, and found that both apoptosis and autophagy occurred in the thymus of piglets infected with HP-PRRSV. In addition to a few virus-infected cells, CD14^+^ cells, the main autophagic cells in the thymus were thymic epithelial cells. These findings demonstrated that HP-PRRSV induces apoptosis in bystander cells, and induces autophagy in both infected and bystander cells in the thymus of infected piglets. Herein, we first present new data on the thymic lesions induced by HP-PRRSV, and show that apoptosis and autophagy are key mechanisms involved in cell survival and determinants of the severity of thymic atrophy in infected piglets. Finally, future studies of the mechanism underlying immune responses are proposed based on our current understanding of PRRSV-host interactions.

## Introduction

Porcine reproductive and respiratory syndrome virus (PRRSV), a small, enveloped, positive single-stranded RNA virus that belongs to the family Arterivirus, is the causative agent of PRRS [1,2]. PRRS causes significant economic losses in the swine industry worldwide, and is characterized by acute respiratory disease in piglets and severe reproductive failure in sows. PRRS and highly pathogenic PRRS (HP-PRRS) emerged in China in 1996 and 2006, respectively [3–6]. Currently, PRRS and HP-PRRS still present health problems to the swine industry worldwide.

Apoptosis, a physiological mechanism of type I programmed cell death, which is important for the development and tissue homeostasis [7,8]. The apoptosis process is controlled by a series of caspases which are regulated through several steps by different mechanisms, and generally, the activation of caspases-3 and -8 are considered to be hallmarks of apoptosis [9–11]. Apoptosis also occurs during viral infections and there is mounting evidence that it can contribute directly to viral pathogenesis [12]. The apoptosis induced by PRRSV has been thought to be a general phenomenon, which can be observed in different organs (thymus, lymph nodes, lungs, and testes) in vivo and in various cell lines in vitro [13–18]. Previously, we demonstrated that HP-PRRSV HuN4 induced the apoptosis of various types of cells in the thymus and peripheral immune organs, and that the apoptosis induced by HP-PRRSV is more severe than that induced by classical PRRSV [19,20]. In the thymus of HP-PRRSV-infected piglets, apoptotic cells were not infected by HP-PRRSV, and most were CD3^+^ T cells. No apoptosis could be observed in epithelial cells, and only a few CD14^+^ cells were apoptotic [21].

Autophagy, a process described as type II programmed cell death, is a series of biochemical events that involve the degradation of unnecessary or dysfunctional cellular components through the actions of lysosomes [22,23]. Autophagy and apoptosis each have distinct mechanisms and pathways. In addition to cellular homeostasis, autophagy is also important for physiological and pathological processes. A hallmark of autophagy is the formation and maturation of autophagosomes, which requires the activities of two ubiquitin-like molecules, microtubule-associated protein 1 light chain 3 (LC3) and Atg12p. LC3 is the most widely used molecular marker to monitor autophagy [22–24]. Recent studies have reported that some viruses can escape host cell autophagy or utilize autophagy for their own replication. For PRRSV, studies have shown that autophagy can be induced in permissive MARC-145 cells and primary pulmonary alveolar macrophages (PAMs) upon PRRSV infection, which can enhance virus replication in vitro [25–27]. However, whether autophagy occurs in PRRSV-infected piglet host cells remains unclear.

Apoptosis involves a series of caspases activity, which leads to cellular structures and organelles demolition in a few minutes [28,29]. Autophagy occurs independently of caspases activity and may be induced when apoptosis pathway is inhibited, thereby eliminating supernumerary or damaged organelles [30–32]. Autophagy and apoptosis might be triggered by common upstream signals, which sometimes results in a combined autophagy and apoptosis. PRRSV HuN4 strain (GenBank accession no. EF635006) is one of the highly pathogenic PRRSV strains, which can cause high morbidity (50–100%), high mortality (20–100%) and the serious injuries in organs in infected-pigs of all ages [6]. Herein, we characterized apoptosis and autophagy in the thymus of piglets infected by the HP-PRRSV HuN4 strain. We found that most autophagic cells are thymus epithelial cells. Our findings demonstrated that both apoptosis and autophagy occurred in the thymi of piglets infected with HP-PRRSV. In contrast to a previous report that PRRSV only induced infected cells to undergo autophagy to facilitate viral replication in vitro, HP-PRRSV can induce autophagy in both infected cells and bystander cells in the thymus of infected piglets.

## Material and Methods

### Animal experiments

A total of twelve 28-day-old PRRSV-negative piglets (Duroc× Landrace× Yorkshire× crossbred) were randomly divided into two groups and were housed separately in isolated rooms. Animals were given commercial feed and water at libitum throughout the experiment. After allowing one week for acclimation, one group of piglets was inoculated with 3 mL HP-PRRSV HuN4 (10^5.5^ TCID_50_ in 3 mL DMEM medium), the other group of piglets was sham-inoculated with 3 mL DMEM medium. Three piglets were humanely euthanized at 7 and 10 days post-inoculation (dpi) from each group. Animal experiments were approved by Animal Ethics Committee of Harbin Veterinary Research Institute of the Chinese Academy of Agricultural Sciences (CAAS) and performed in accordance with animal ethics guidelines and approved protocols. The Animal Ethics Committee approval number was SYXK (Hei) 2014015.

### Analysis of apoptotic changes in thymus

Thymic cells apoptosis and necrosis were evaluated by flow cytometry analysis, as described previously [33], using the Annexin V-FITC Apoptosis Detection Kit I (BD Biosciences; Franklin Lakes, NJ, USA) according to the manufacturer’s instructions.

### Western blotting

Thymus samples were washed with PBS three times, incubated on ice with cell lysis buffer (50 mM Tris-HCl, pH 7.4, 150 mM NaCl, 1% Triton X-100, 2 mM EDTA, 0.1% SDS, and 5 mM sodium orthovanadate) containing a protease inhibitor cocktail (04693132001; Roche, Bern, Switzerland) and 0.1 mM PMSF for 2 h. Cell lysates were centrifuged at 13,000×*g* for 20 min at 4°C. Protein concentrations were determined using the Bradford assay (ThermoScientific, Waltham, MA, USA). Equal amounts of protein samples were loaded on 12% (w/v) SDS—PAGE gels. Proteins were transferred from the gels to polyvinylidene fluoride (PVDF) membranes (ISEQ00010, Millipore, Billerica, MA, USA) and then were blocked with 5% dry milk dissolved in PBS-T (PBS in 0.05% Tween 20, pH 7.4) at 4°C overnight. Membranes were incubated with primary antibodies (anti-LC3 antibody and anti-P62 antibody, 1:1000, Sigma; anti-caspase-3 antibody and anti-caspase-8 antibody, 1:500, Cell Signaling; anti-β-actin antibody, 1:1000, TransGen Biotech) for 1 h at room temperature (RT) and then were incubated at 4°C overnight. After washing with PBS-T, either DyLight 800-labeled goat anti-rabbit antibody or DyLight 800-labeled goat anti-mouse antibody (1:5000, Kirkegaard & Perry Laboratories, Gaithersburg, MD, USA) was added and incubated for 2h at RT. Blots were developed at an appropriate excitation wavelength using a digital imaging system (Odyssey infrared imaging system; LI-COR Biosciences, Lincoln, NE, USA).

### Confocal microscopy

During necropsy, thymus samples were collected and sectioned (8 μm) on a cryostat. The cryostat sections were used for double-immunofluorescence staining. To examine whether PRRSV colocalized with apoptotic or autophagic cells, thymus sections were stained with SR30F (1:50, RTI, SD) against the PRRSV N protein along with anti-LC3 antibody (1:100, Sigma), SR30F, or anti-LC3 antibody, respectively. Sections were stained with anti-rabbit secondary antibodies conjugated to tetraethyl rhodamine isothiocyanate (TRITC) or fluorescein isocyanate (FITC) (1:100, ZSGB-BIO, Beijing, China), or were subjected TUNEL staining following the In Situ Cell Death Detection Kit manufacturer’s instructions (Roche). Finally, nuclei were stained with 4′-6-diamidino-2-phenylindole (DAPI, Invitrogen, Carlsbad, CA, USA). To characterize the types of cells that underwent autophagy, thymus sections were stained with anti-LC3 antibodies followed by anti-rabbit secondary antibodies conjugated to TRITC or FITC. Thymus sections were stained with FITC-conjugated mouse anti-pig CD14 antibody (1:50, AbD Serotec, Oxford, UK) to detect CD14^+^ cells, or spectral red (SPRD)-conjugated mouse anti-pig CD3 antibody (1:50, Southern Biotech, Birmingham, AL, USA) to detect CD3^+^ cells, or mouse anti-pan-cytokeratin (P-CK) antibodies (1:25, Gene Tech, China) and FITC-conjugated goat anti-mouse antibody (1:100, ZSGB-BIO, Beijing, China) to detect thymic epithelial cells. Finally, nuclei were strained with DAPI. Sections were observed using a laser-scanning confocal microscope.

### Statistical analysis

Statistical analysis was carried out using the Student’s t-test.

## Results

### HuN4 induces apoptosis in thymic cells from HuN4-infected piglets

**Fig 1:**
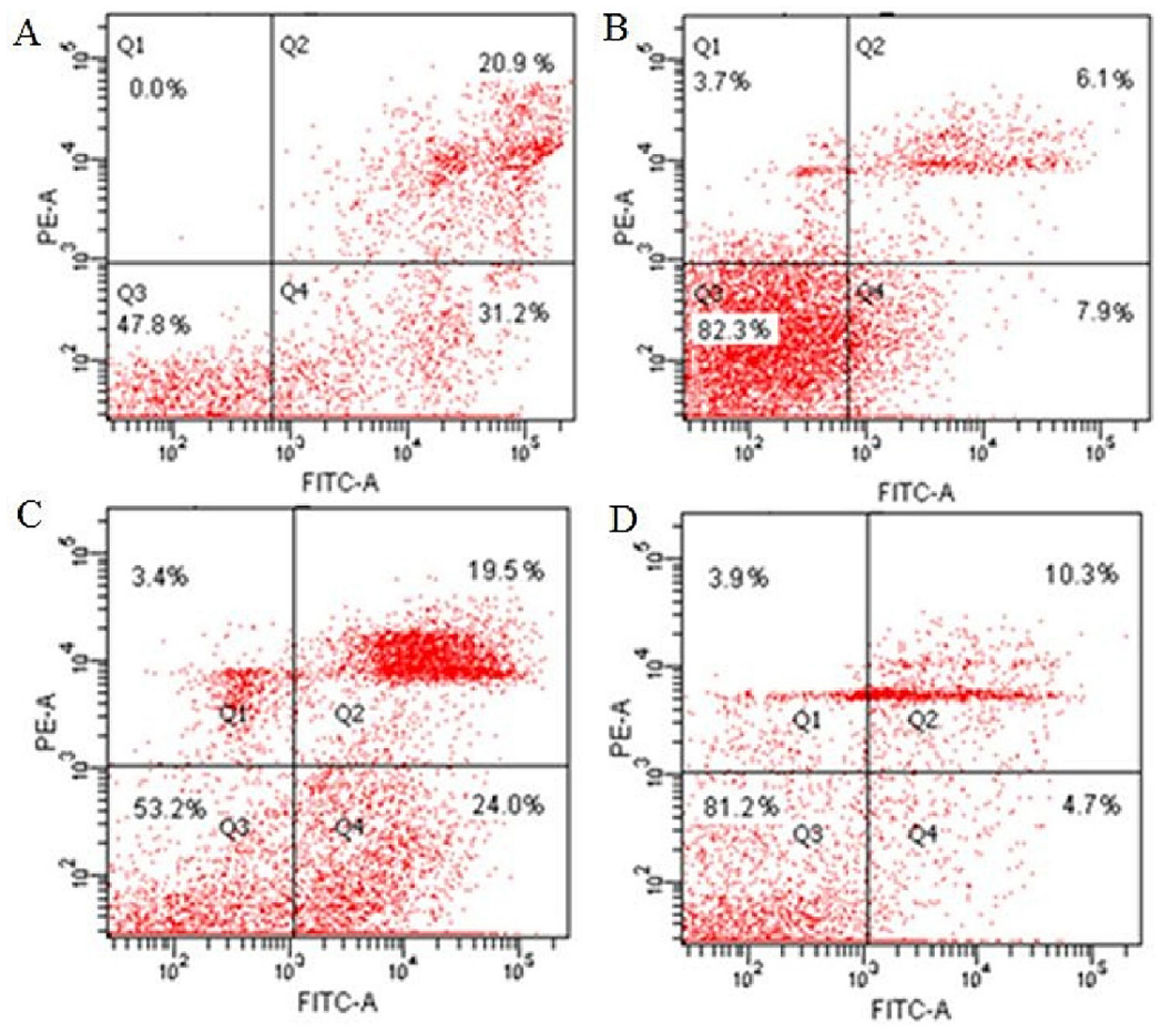
The kinetics of thymocytes apoptosis at different days post-infection (dpi). The percentages of apoptotic cells in the thymus of one representative HuN4-infected piglet at 7 (A) and 10 (C) dpi, and apoptotic cells in the thymus of a representative age-matched control piglet at 7 (B) and 10 (D) dpi. Apoptotic cells were quantified by flow cytometry using FITC-labeled Annexin V. Q1, necrotic or another cell population that was FITC-Annexin V-negative and PI-positive; Q2, end stage apoptotic or a dead cell population that was FITC-Annexin V- and PI-positive; Q3, a viable cell population that was not undergoing apoptosis and was both FITC-Annexin-V- and PI-negative; Q4, an early apoptotic cell population that was FITC-Annexin V-positive and PI-negative.

Apoptotic changes in thymi from experimental piglets were investigated using flow cytometry. The frequency of apoptotic cells in thymi from the HuN4-infected group was ~50% and ~45% at 7 or 10 dpi, respectively (Fig 1). During the experiment, the percentage of apoptotic cells in the thymi of control group piglets was ~14%.

### HuN4 induces caspase-dependent apoptosis in thymic cells from infected piglets

**Fig 2:**
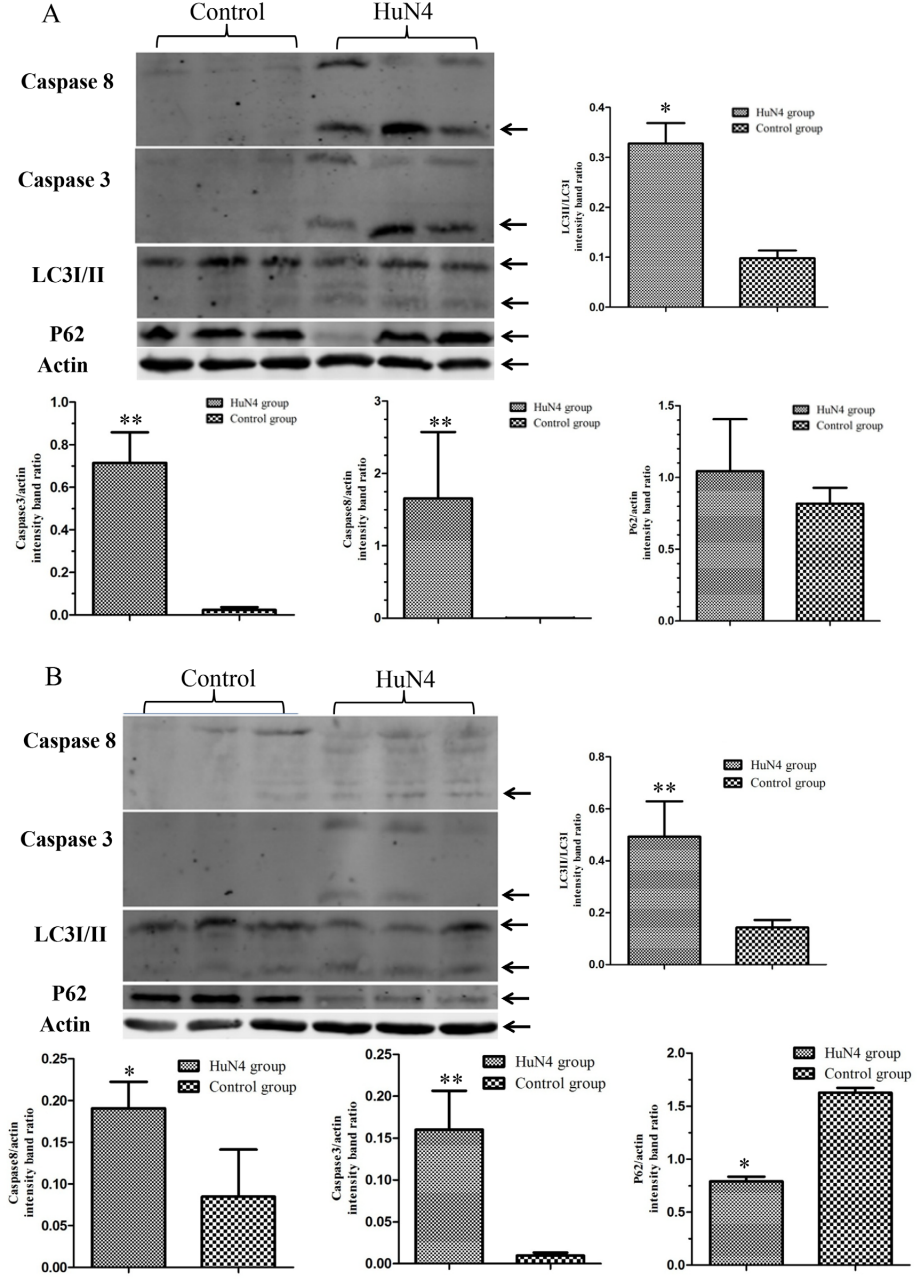
Apoptosis and autophagy are induced by HuN4 infection in the thymus of infected piglets. Levels of proteins related to apoptosis or autophagy in the thymus of piglets at 7 (A) and 10 (B) dpi. Thymus tissues were lysed and western blots were performed. Western blotting results were analyzed digitally and the optical density ratio was calculated, and the bands which were in use for densitometry marked by the arrows; the ratio representatives the mean of measurements for piglets at different time points and the Student’s t-test were carried for the statistical analysis. (n = 3 per time point).

The activation of caspases-3 and -8 are considered to be hallmarks of apoptosis, so we investigated the cleavage of caspases-3 and -8 in thymic cells by western blot. We found that HuN4 viral infection induced the cleavage of caspase-3 and caspase-8 from 7 dpi (Fig 2A), which persisted at 10 dpi (Fig 2B). In thymi from control piglets, one of three piglets showed caspase-8 bands similar to the infected piglets at 10dpi. Because thymocytes apoptosis also happens in the thymi of control piglets, compared to 7 dpi, the percent of the early apoptotic cell in the thymi of infected piglets become lower (Fig 1C), and the activation of caspase-8 pathway mild and may have the similar result with the thymi of control piglets. These findings further indicated the occurrence of apoptosis in thymi from HP-PRRSV-infected piglets.

### HuN4 induces autophagy in thymic cells from infected piglets

Whether PRRSV infection triggers autophagy in thymi was investigated by detecting modified LC3. Thymi from infected and control piglets were collected at 7 and 10 dpi and western blotting was performed using an anti-LC3 polyclonal antibody that recognizes both LC3-I and LC3-II. We found that LC3-I was converted to its lipidated form LC3-II, in thymi from HuN4-infected piglets from 7 dpi (Fig 2A), and densitometric analysis using Gene Tools also showed that the LC3-II/LC3-I ratio significantly increased at 7 and 10 dpi. Changes in an LC3-dependent ubiquitinated substrate, p62, were also detected by western blotting. In thymi from HuN4-infected piglets, only one of three piglets showed reductions in ubiquitinated p62 at 7 dpi (Fig 2A); however, thymi from all three HuN4-infected piglets showed obviously reduced levels at 10 dpi (Fig 2B).

### Associations between HuN4-infected cells, apoptotic cells, and autophagic cells

**Fig 3:**
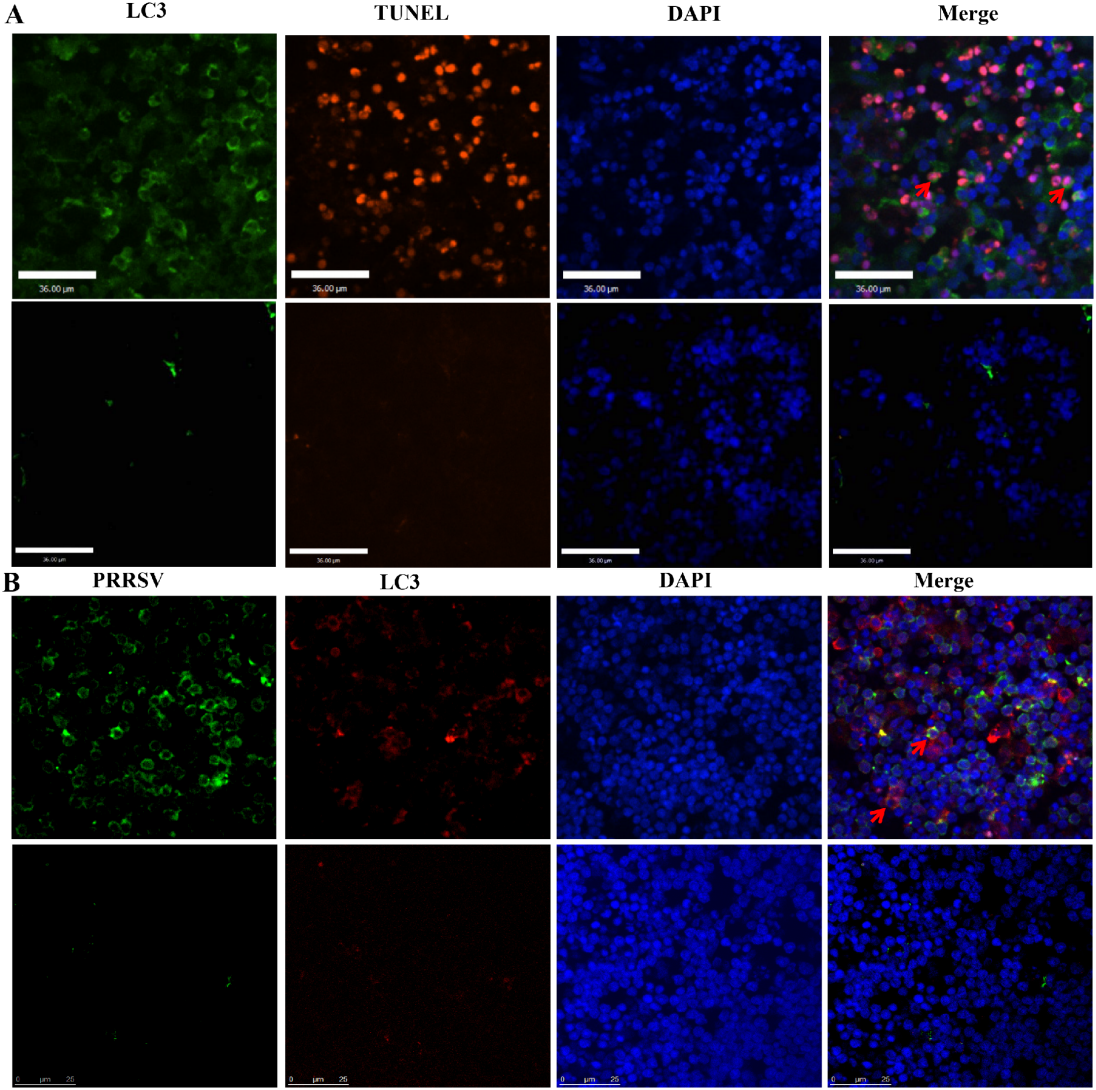
Colocalization of PRRSV-infected cells, autophagic cells, and nuclei. Autophagic cells were stained with anti-LC3 antibodies and anti-rabbit secondary antibodies conjugated to FITC, and apoptotic cells were detected according to the manufacturer’s instructions for the In Situ Cell Death Detection Kit, only a few apoptotic cells underwent autophagy (arrows) (A); Autophagic cells were stained with anti-LC3 antibodies and tetramethylrhodamine- isothiocyanate (TRITC)-conjugated goat anti-mouse antibodies, and HuN4-infected cells were strained with fluorescein isothiocyanate (FITC)-conjugated monoclonal antibodies (mAbs) against PRRSV N protein, and some HuN4-infected cells underwent autophagy (arrows) (B). Nuclei were stained with 4-6-diamidino-2-phenylindole (DAPI).

Double immunofluorescence staining was used to examine whether PRRSV colocalized with autophagic or apoptotic cells. We found that HuN4-infected cells did not undergo apoptosis, which correspond to our previous study [21], only a few apoptotic cells underwent autophagy (Fig 3A), and some HuN4-infected cells underwent autophagy (Fig 3B). These findings demonstrated that HP-PRRSV induces apoptosis in bystander cells, and can induce autophagy in both infected and bystander cells in thymi of infected piglets.

### Characterization of cells undergoing autophagy

**Fig 4:**
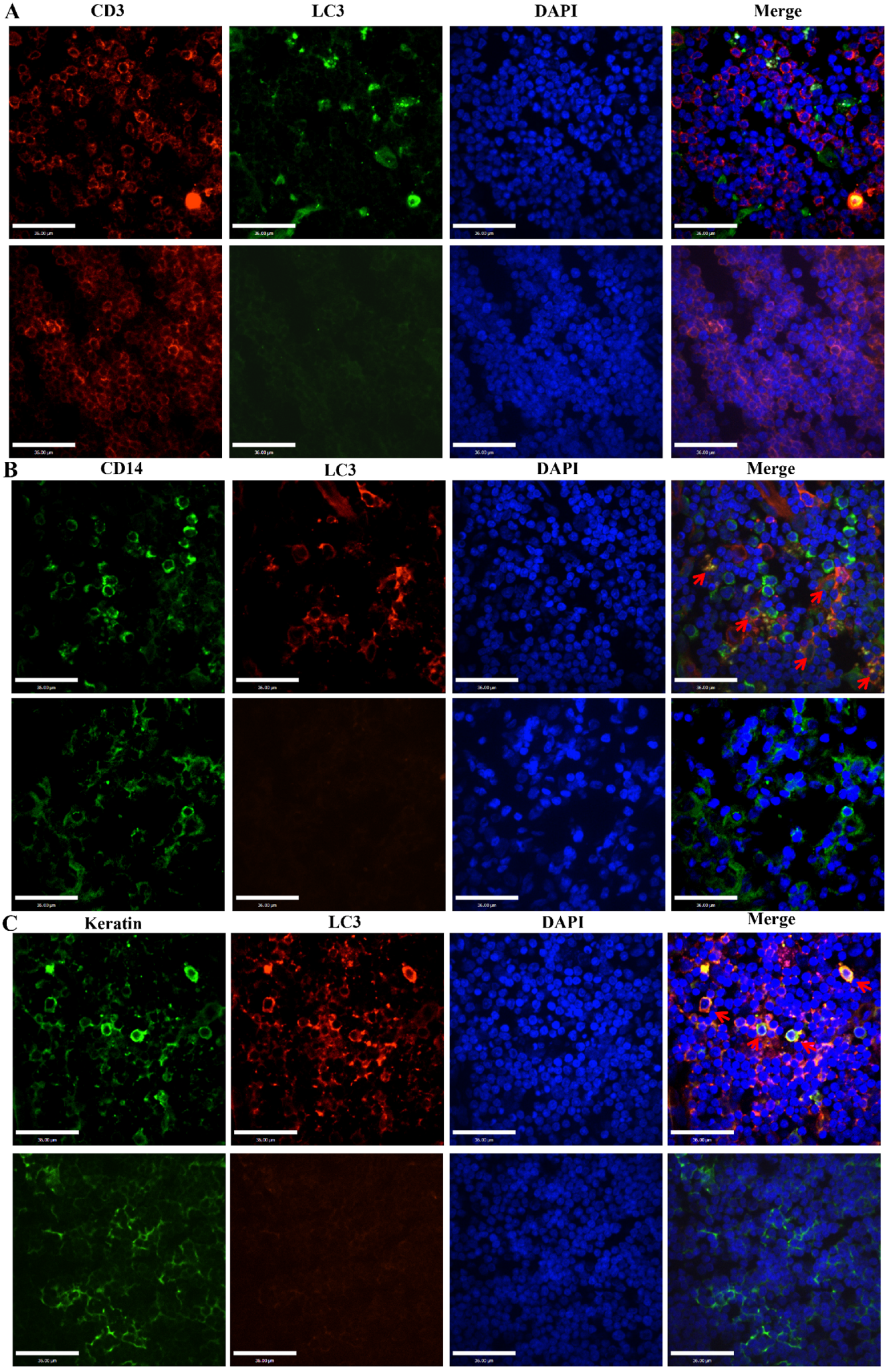
Colocalization of CD3^+^ cells, CD14^+^ cells, thymic epithelial cells, and autophagic cells. CD3^+^ cells were stained with spectral red (SPRD)-conjugated mouse anti-pig CD3 antibodies, and autophagic cells were stained with anti-LC3 primary antibodies and anti-rabbit secondary antibodies conjugated to FITC, no CD3^+^ cells underwent autophagy (A); CD14^+^ cells were stained with FITC-conjugated mouse anti-pig CD14 antibodies and autophagic cells were stained with anti-LC3 primary antibodies and TRITC-conjugated goat anti-mouse antibodies, some CD14^+^ cells underwent autophagy (arrows) (B). Thymic epithelial cells were stained with mouse anti-pan-cytokeratin (P-CK) antibodies and FITC-conjugated goat anti-mouse antibodies, and autophagic cells were stained with anti-LC3 primary antibodies and TRITC-conjugated goat anti-mouse antibodies, most autophagic cells colocalized with thymic epithelial cells (arrows) (C). Nuclei were stained with DAPI.

Previously, we characterized HuN4-infected cells, which were CD14^+^ cells, and apoptotic cells, which were mainly CD3^+^ cells [21]. Here, to characterize the types of thymic cells undergoing autophagy, CD14^+^ cells were also stained with FITC-conjugated mouse anti-pig CD14 antibodies, CD3^+^ cells were stained with spectral red (SPRD)-conjugated mouse anti-pig CD3 antibodies, and thymic epithelial cells were stained with mouse anti-pan-cytokeratin (P-CK) antibodies and FITC-conjugated goat anti-mouse antibodies. LC3 was strained using both anti-LC3 polyclonal antibody and FITC/TRITC-conjugated goat anti-rabbit antibodies. We found that no CD3^+^ cells underwent autophagy (Fig 4A), most autophagic cells colocalized with thymic epithelial cells (Fig 4C), and only some cells that underwent autophagy colocalized with CD14^+^ cells (Fig 4B).

## Discussion

The HP-PRRSV HuN4 strain can cause severe thymic atrophy in infected piglets after birth [19,34]. The number of apoptotic thymocytes might be one factor that leads to thymic atrophy. Herein, we investigated whether thymocytes also underwent autophagy during HuN4 infection, which might be another factor related to thymus atrophy induced by HP-PRRSV.

As an important primary lymphoid organ, the thymus contains thymocytes at various stages of T cell differentiation and maturation [35]. Upon HP-PRRSV infection, viruses induce the apoptosis of uninfected bystander CD3^+^ thymocytes, a mechanism that likely contributes to the loss of CD3^+^ thymocytes and affects immature T cell development. To date, little is known about the role of apoptosis in uninfected bystander cells in the thymus of infected piglets. In thymus of HuN4 infected-piglets, infected cells maybe secrete proapoptotic cytokines or deliver apoptotic signals to thymocytes undergoing selection for self-recognition, as HP-PRRSV induced the robust production of proinflammatory cytokines, especially IL-1β, IL-6, and TNF-α [36,37].

Previous studies showed that PRRSV can induce autophagy in permissive MARC-145 cells and primary pulmonary alveolar macrophages (PAMs) upon PRRSV infection in vitro [25–27]. Those findings showed that PRRSV induced infected cells to undergo autophagy in order to enhance replication. In this present study, in addition to virus-infected CD14^+^ cells, many HP-PRRSV-negative thymic epithelial cells also underwent autophagy (Fig 4). This finding is different from previous reports that PRRSV only induced infected cells to undergo autophagy to allow viral replication in vitro [25–27]. The reason maybe the role of autophagy in vivo is more complex than in vitro, the autophagy pathway serves the main function of degrading large protein complexes, it also has additional functions in the innate and adaptive immune response [38] in vivo. In thymus, thymic epithelial cells play an important role in the positive and negative selection of thymocytes, which occurs via interactions with antigens presented by MHC complexes on the cell surface [35]. Autophagy has been proposed to have an essential function in the thymic epithelium, which compartmentalizes access to the MHC class II complexes in the thymus, and consequently plays an important role in presenting self-Ags to developing/immature thymocytes by intersecting with the MHC class II presentation pathway [39]. In this present study, during the course of HP-PRRSV infection in the thymus, complex crosstalk between autophagy and apoptosis occurs. Autophagy can represent a stress adaptation that allows cells to avoid death, or in other cellular settings can constitute an alternative cell-death pathway [40,41]. Autophagy and apoptosis can sometimes be triggered by common upstream signals, which can result in both autophagy and apoptosis. The functional relationship between autophagy and apoptosis can be complex under certain circumstances. In other instances, cells can switch between the two responses in a mutually exclusive manner [42]. Apoptotic cell death can be induced by inhibiting the accumulation of autophagosomes in various carcinoma cells [43], suggesting that autophagy prevents apoptotic cell death. In this present study, whether autophagy occurs during conditions of severe thymus atrophy to ‘educate’ thymocytes and regenerate the T cell compartment with immunocompetent cells-to compensate for the decreased number of CD3^+^ cells—remains unclear. The relationship between autophagy and the many apoptotic thymocytes in thymi of HP-PRRSV-infected piglets remains to be investigated.

In conclusion, the HP-PRRSV HuN4 strain caused severe thymic atrophy in infected piglets after birth, which was related to a high frequency of thymocyte apoptosis and autophagy. HP-PRRSV induces apoptosis in CD3^+^ cells, and induces autophagy in both infected CD14^+^ cells and bystander cells- thymic epithelial cells in the thymus of infected piglets.
